# Spatiotemporal Regulation of Rho GTPases in Neuronal Migration

**DOI:** 10.3390/cells8060568

**Published:** 2019-06-10

**Authors:** Zhenyan Xu, Yuewen Chen, Yu Chen

**Affiliations:** 1 The Brain Cognition and Brain Disease Institute, Shenzhen Institutes of Advanced Technology, Chinese Academy of Sciences, Shenzhen-Hong Kong Institute of Brain Science-Shenzhen Fundamental Research Institutions, Shenzhen 518055, Guangdong, China; zy.xu2@siat.ac.cn (Z.X.); yw.chen1@siat.ac.cn (Y.C.); 2 Guangdong Provincial Key Laboratory of Brain Science, Disease and Drug Development, HKUST Shenzhen Research Institute, Shenzhen 518057, Guangdong, China

**Keywords:** Rho GTPase, guanine nucleotide exchange factor, GTPase-activating protein, neuronal migration, neuronal development

## Abstract

Neuronal migration is essential for the orchestration of brain development and involves several contiguous steps: interkinetic nuclear movement (INM), multipolar–bipolar transition, locomotion, and translocation. Growing evidence suggests that Rho GTPases, including RhoA, Rac, Cdc42, and the atypical Rnd members, play critical roles in neuronal migration by regulating both actin and microtubule cytoskeletal components. This review focuses on the spatiotemporal-specific regulation of Rho GTPases as well as their regulators and effectors in distinct steps during the neuronal migration process. Their roles in bridging extracellular signals and cytoskeletal dynamics to provide optimal structural support to the migrating neurons will also be discussed.

## 1. Introduction

The ability of the neocortex to control complex cognitive and motor tasks is dependent on a well-organized six-layered architecture, which arises from a remarkable process called neuronal migration. In neuronal migration, neurons originating from neuroepithelial or radial glial progenitors (RGPs) in the ventricular zone (VZ) and subventricular zone (SVZ) migrate a long distance to their final destinations [1,2]. In this review, due to space limitations, we focus on the radial migration of projection neurons in the developing cortex. The role of Rho GTPases in the migration processes in the adult brain and the tangential migration adopted by most interneurons have been recently reviewed elsewhere [3,4].

At the beginning of neurogenesis, neuroepithelial progenitor cells expand and the preplate forms. At this stage, RGPs elongate their processes from the ventricular side to the pial brain surface. Then, RGPs generate most of the neurons by asymmetric division through interkinetic nuclear migration (INM). The newly born neurons in the intermediate zone (IZ) have a multipolar morphology with dynamic leading and trailing processes, and convert to a bipolar morphology following a transient stationary phase. These bipolar neurons migrate out of the IZ into the cortical plate (CP), guided by the basal processes of RGPs. Earlier-born neurons occupy the lower lamination, and the later-born neurons settle at the appropriate position above the lamination generated by the earlier-born neurons. Thus, the six-layered neocortex is established through this inside-out mode of development.

Both the actin and microtubule cytoskeleton are essential for coordinating the steps of neuronal migration. RGPs are highly polarized along the apical–basal axis and form adherens junctions with neighboring RGPs. The destruction of adherens junctions results in defects in radial glial scaffolding and radial glia-guided locomotion [5]. In addition, the multipolar–bipolar transition of neurons in the IZ requires the participation of the cytoskeleton, which helps establish contacts with RGPs for guidance [6]. After adhering to RGPs during the journey from the IZ to the CP, a leading process extends at the leading end of migrating neurons, a cytoplasmic dilation is formed at the proximal region, and the microtubule network reorganizes to enmesh the nucleus and promote nuclear elongation and forward movement coupled with the actin cytoskeleton. When the leading process reaches the marginal zone, neurons uncouple from RGPs and undergo terminal translocation to their final destination [7,8,9].

Rho GTPases are a part of the Ras superfamily and play an important role in neuronal migration by coordinating cytoskeletal dynamics [10]. There are several classic Rho GTPases including Rac1, Cdc42, and RhoA. They cycle between an active GTP-bound form and an inactive GDP-bound form and thus coordinate cytoskeletal dynamics in various subregions of RGPs or migrating neurons [11]. Their activity can be augmented by Rho-specific guanine nucleotide exchange factors (GEFs) and inhibited by GTPase-activating proteins (GAPs). Besides classic Rho GTPases, the atypical Rnd members are also involved in neuronal migration. However, unlike classic Rho GTPases, they maintain constitutive activity, and their actions can be regulated primarily by their expression level [12,13,14,15,16]. In this review, we will discuss the spatiotemporal regulation of Rho GTPases and their regulators in neuronal migration. 

## 2. Interkinetic Nuclear Migration in the Cortical Ventricular Zone

**Figure 1:**
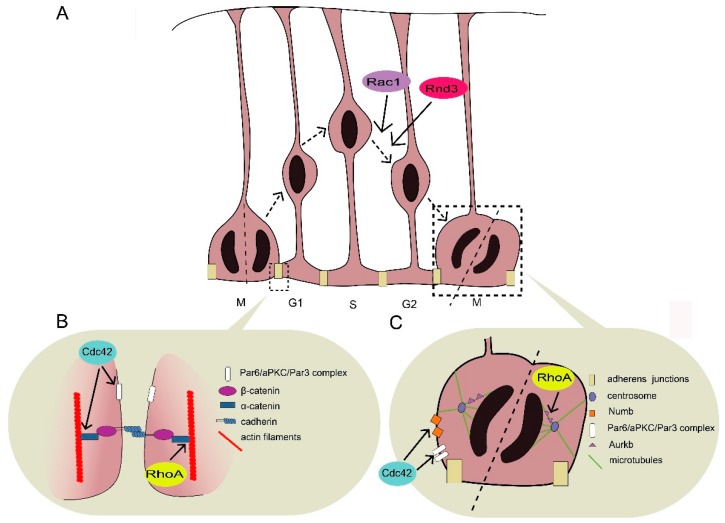
Rho GTPases regulate neurogenesis or interkinetic nuclear migration (INM). (**A**) Radial glial progenitors (RGPs) line the ventricular surface and exhibit apical–basal polarity. The nuclei within RGPs undergo an oscillatory form of cycle-dependent migration, which leads to symmetric or asymmetric cell division at the ventricular surface; the former expands the RGP pool, and the latter generates basal progenitors or neurons. Rac1 and Rnd3 promote basal–apical migration. (**B**) The proper structure of RGPs depends on the maintenance of adherens junctions and cell polarity, which are regulated by Cdc42 and RhoA. Adherens junctions directly link adjacent RGPs via junctional complex, which is a quaternary complex comprising cadherin, β-catenin, α-catenin, and actin filaments. Both Cdc42 and RhoA may help tension the link between α-catenin and actin filaments. In addition, Cdc42 contributes to maintaining cell polarity through Par6/aPKC/Par3 complex. (**C**) Besides cell structure, Cdc42 and RhoA help balance the proliferation and differentiation of RGPs. RhoA primarily regulates mitotic spindle orientation and cytokinesis through Aurora kinase B (Aurkb). Cdc42 mainly promotes self-renewing ability through regulating the apical localization of Numb and Par6/aPKC/Par3 complex.

Immediately after neural tube closure, neuroepithelial cells, which line the ventricular surface, elongate their basal process and transform into RGPs [17]. The nuclei within RGPs undergo an oscillatory form of cycle-dependent migration termed interkinetic nuclear migration (INM) [18] (Figure 1A). The INM of RGPs leads to symmetric or asymmetric cell division at the ventricular surface. The former expands the RGP pool, while the latter yields post-mitotic neurons or intermediate progenitors, which subsequently generate neurons [17]. In addition to neurogenesis, RGPs serve as a scaffold for neuronal migration through their long parallel processes.

RGPs have a unique cellular architecture characterized by apical–basal polarity and adherens junctions between neighboring cells lining the ventricular surface and the marginal zone via pial surface end-feet. The maintenance of cell–cell adhesion and the integrity of the VZ are critical for the construction of a scaffold for neuronal migration [5]. In addition, the intact organization of the RGPs maintains homeostasis between proliferation and differentiation. Once separated from the VZ, RGPs exhibit a preference towards hyperproliferation rather than differentiation [19]. Therefore, it is important for RGPs to sustain a proper architecture in developing tissues, because a series of biological events is likely to occur in a spatial-specific manner in the proper order.

Although the molecular details by which Rho GTPases regulate cell proliferation and cell fate in RGPs during INM are not well characterized, evidence indicates that Rho GTPases affect RGP organization, proliferation, and cell-fate specification by regulating the cytoskeleton and thus the distribution and localization of cell fate-determinate proteins such as Numb [20,21].

### 2.1. Maintenance of RGP Structrure

Adherens junctions directly link adjacent RGPs with actin filaments (Figure 1B). The junctional complex is usually considered a quaternary complex of cadherin, β-catenin, α-catenin, and actin [22,23]. Adherens junctions initiate the formation of this complex via actin assembly at the plasma membrane, followed by the formation of protrusions, and finally the assembly of adhesion proteins such as cadherins and catenins [22,23]. Adherens junctions participate in building communication among the coupling RGPs. Several studies show that adherens junctions are required to maintain the integrity of the VZ and that disruption of cell–cell adhesion leads to alterations of cell signaling pathways that regulate neural development [24,25,26,27,28,29,30]. The disruption of adherens junctions results in various migration disorders including cortical hypoplasia and subcortical band heterotopia (SBH, also called double cortex), which result from the dysregulation of Cdc42 and RhoA, respectively [30,31,32,33,34]. In addition, the end-feet of RGPs, which tie basal processes with pial surface, are important for the migration process, as disruption of end-feet leads to pial basement membrane impairment, which can further lead to cobblestone lissencephaly [35,36,37,38].

#### 2.1.1. Role of Cdc42 in RGP Structure Maintenance

In the developing cerebral cortex, Cdc42 is expressed at the apical side of RGPs. Its activity and apical localization are regulated by kinases such as myristoylated alanine-rich C kinase substrate (MARCK) [39]. Several studies suggest that Cdc42 deletion can lead to the delocalization of E-cadherin and β-catenin—the constituent proteins of adherens junctions—leading to adherens junction impairment and cortical hypoplasia [30,32,34]. In addition, Cdc42 deletion abolishes the apical localization of other proteins including Par6 and atypical protein kinase C (aPKC), which subsequently results in the disruption of adherens junctions [24,28,32,34,40]. Moreover, the polarity module formed by Cdc42/Par6/aPKC/Par3 appears to be essential for apical polarity establishment, suggesting that active GTP-bound Cdc42 can recruit and activate a cytoplasmic Par/aPKC complex and thus help induce apical polarity, which is critical for adherens junction formation [41]. 

Cdc42 also accumulates in the basal end-feet to regulate the cytoskeleton via the Arp2/3 complex and thus maintains the structure of the basal end-feet [19]. Cdc42 deficiency results in the impairment of nestin-positive RGP fibers in the cortex, which leads to the failure of attachment to the pial surface [34]. 

#### 2.1.2. Role of RhoA in Adherens Junction Formation and Maintenance

RhoA activity in RGPs can be regulated by Rho-specific GEFs such as ArhGEF18 and Lfc [25,26]. In normal embryos, RGPs align themselves at the ventricular surface. However, genetic deletion of RhoA results in a scattered distribution of RGPs all around the cerebral cortex, together with disrupted cadherin/catenin complex and cadherin-based adherens junctions [27,29,33]. Mammalian diaphanous-related formin1 (mDia1), a downstream effector of RhoA localized at the ventricular surface of adherence junctions, is reduced upon RhoA deletion, thus destabilizing the cytoskeleton and junction structure [27]. In addition, another downstream effector of RhoA, Rho-associated protein kinase 2 (ROCK2), helps organize adherens junctions in the medaka fish [26]. Both mDia1 and ROCK2 promote actin stress fiber formation. Although it is unknown whether mDia1 or ROCK2 is the main supporter of stress generation in RGPs in the rodent cortex, these results suggest that sufficient physical tension mediated by RhoA is important for maintaining adherens junctions. Moreover, RhoA deletion in neural progenitor cells gradually disrupts adherens junctions from mouse embryonic day 11.5–14.5, while Cdc42 deletion results in increased basal mitosis and neuronal numbers immediately from embryonic day 9.5–10.5 [29,30,33,34]. This indicates that Cdc42 and RhoA affect different aspects of adherens junction formation and maintenance, although their deletion similarly leads to the disruption of adherens junctions.

### 2.2. Balance between RGP Proliferation and Differentiation

To ensure proper cortical development, the number of cortical neurons formed is determined by a strictly controlled balance between the expansion of the RGP pool and neurogenesis (Figure 1C). When the RGPs lining the ventricular surface receive aberrant extracellular signals from the ventricular fluid or neighboring cells, this perturbs adherens junctions and causes an imbalance between proliferation and/or differentiation rates [42]. Cell fate is also regulated by intrinsic mechanisms. For instance, during asymmetrical division, the daughter cell expressing Numb protein preferentially remains apical as a progenitor cell [21]. Dysregulation of these factors usually leads to imbalanced proliferation and differentiation. For example, hyperproliferation of RGPs results in exencephaly, while premature differentiation or inadequate proliferation leads to brain hypoplasia such as holoprosencephaly or microcephaly [30,31,32,33,34,43,44].

#### 2.2.1. RhoA Deletion Results in Abnormal RGP Proliferation

In the developing cortex, RhoA deficiency retains RGPs in a proliferative state, which delays neurogenesis and results in an increased number of mitotic cells, expansion of the RGP pool, and exencephaly [31,33]. One possible explanation for this phenomenon is that signaling pathways that downregulate proliferation are impaired when RGPs leave the ventricular surface owing to the disruption of adherens junctions upon RhoA depletion [19,28,29]. For example, β-catenin accumulates in the nucleus to activate proliferation when the adhesion formed by adherens junctions is disturbed [19]. Alternatively, the delayed neurogenesis can be the result of aberrant mitotic spindle orientation caused by RhoA deletion. Aurora kinase B (Aurkb) is a downstream target of RhoA that is implicated in cytokinesis and the vertical orientation of the cleavage plane. Accordingly, inhibition of RhoA leads to the delocalization of Aurkb during mitosis [45]. Moreover, in the absence of RhoA, the hedgehog signaling pathway is upregulated, which has been reported to promote RGP hyperproliferation [29,46]. However, it is unknown how RhoA cooperates with the hedgehog signaling pathway to regulate the cell cycling of RGPs. Nevertheless, these studies strongly support the idea that RhoA plays an important role in the cell cycle regulation of RGPs.

#### 2.2.2. Cdc42 Deletion Results in Reduced RGP Self-Renewal

In contrast to RhoA deletion, Cdc42 deficiency leads to accelerated cell-cycle exit and enhanced neurogenesis [30,32,34,43,47]. Loss-of-function studies show that depletion of Cdc42 in neural progenitor cells results in INM failure characterized by increased neuron number, defective RGP fibers, and holoprosencephaly [30,32,34]. Cdc42 can form Cdc42/Par6/aPKC/Par3 complex to help stabilize adherens junctions. Intriguingly, loss of Par polarity proteins such as aPKC results in the disappearance of adherens junctions and disruption of ventricular surface integrity but does not contribute to defective RGP proliferation that leads to holoprosencephaly [28]. The difference in the severity of brain malformations between polarity protein deletion and Cdc42 deletion indicates that Cdc42 not only plays a central role in adherens junction formation, but is also necessary for the maintenance of the specific fate of self-renewing RGPs [28,32,34]. In the absence of Cdc42, the apical localization of Numb is impaired, leading to the loss of the self-renewal ability of RGPs [21,30,32,34]. In addition, once RPGs leave the VZ owing to Cdc42 deficiency, some marker proteins of basal progenitors (e.g., Ngn2, Tbr2, NeuroD1, Svet1, and VGlut2) are upregulated. Therefore, these RPGs exhibit characteristics tending toward differentiation rather than self-renewal [32]. However, the mechanistic action by which Cdc42 controls the balance between the proliferation and differentiation of RGPs needs further characterization.

#### 2.2.3. Rac1 Deletion Results in Accelerated Cell-Cycle Exit

In the VZ, Rac1 is enriched on the apical side in RGPs on the ventricular surface [47]. Rac1 deletion leads to a gradual reduction of the RGP pool in the developing cortex and microcephaly primarily due to accelerated cell-cycle exit and increased apoptosis [43,44]. However, the reduction of RGPs in the cortex upon Rac1 deletion appears at about embryonic day 17.5, which is much later than that upon Cdc42 deletion [33,34,43,44]. In addition, Rac1 deletion reduces proliferative cells and increases differentiated cells in the SVZ where basal progenitors localize [44]. The accelerated cell-cycle exit of basal progenitors may contribute to the reduced forebrain size. The aberrant cell-cycle regulation upon Rac1 deletion may be partly due to the downregulation of Cyclin D2, which is required for SVZ cell population expansion [44]. These studies suggest that Rac1 deletion primarily impairs the self-renewal ability of both RGPs and basal progenitors at a later neurogenesis stage. 

### 2.3. Rac1/Rnd3 Deletion Results in Abnormal INM

In contrast to Cdc42 deletion, loss of Rac1 does not affect cell polarity or adherens junction formation but disturbs basal–apical nuclear movement (i.e., G2 phase) during INM [47]. The impaired nuclear movement suggests that the microtubule or actomyosin cytoskeleton, which are responsible for nuclear migration, might be disrupted in the absence of Rac1 [48]. Interestingly, dedicator of cytokinesis 7 (DOCK7), a member of DOCK family RacGEFs, regulates basal–apical nuclear movement and neurogenesis by antagonizing a centrosome-associated protein, transforming acidic coiled-coil-containing protein 3 (TACC3), rather than modulating Rac1 activity [2,49,50].

Besides the classic Rho GTPases, Rnd3 (an atypical Rho GTPase) is also involved in the INM process. The basal–apical nuclear movement is delayed upon loss of Rnd3 activity [51]. Through the modulation of the actin cytoskeleton, Rnd3 is involved in the apical attachment of RGPs to the ventricular surface as well as cleavage plane orientation [51]. In addition, Rnd3 inhibits the proliferative activity of basal progenitors by suppressing Cyclin D1 translation [51]. However, the signaling cascades that mediate the action of Rnd3 in RGPs await further exploration. 

## 3. Multipolar–Bipolar Transition

**Figure 2:**
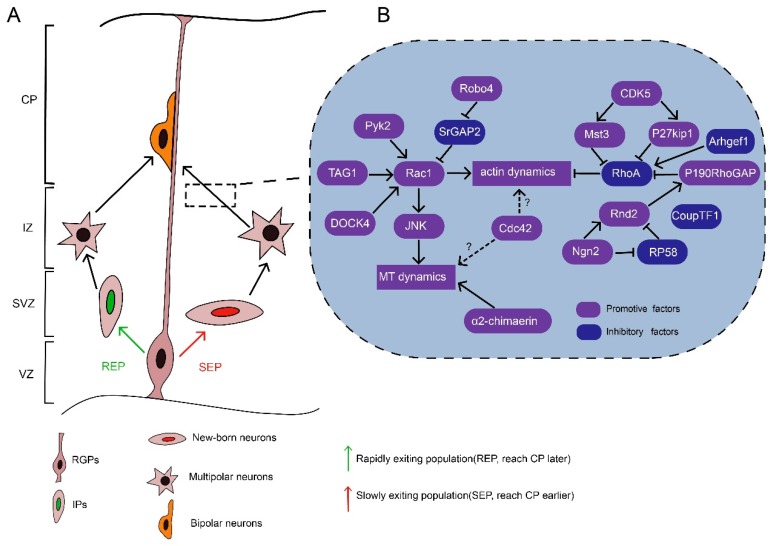
Rho GTPases regulate the multipolar–bipolar transition of nascent neurons. (**A**) There are two differentiation modes by which RGPs become multipolar neurons, which are represented by two populations: the “rapidly exiting population” (REP, green) and the “slowly exiting population” (SEP, red). In the REP route, RGPs generate intermediate progenitors (IPs), which undergo division and differentiate into neurons. In the SEP route, RGPs directly produce nascent neurons that remain in the lower part of the subventricular zone (SVZ). Both SEP and REP neurons acquire a multipolar morphology in lower intermediate zone (IZ) and take on a bipolar morphology when they reach the cortical plate (CP). (**B**) The multipolar–bipolar transition relies on actin and microtubule dynamics, which are under the precise regulation of various promotive factors (purple) and inhibitory factors (blue). Globally, the Rac1 pathway is activated to promote both actin and microtubule dynamics, while RhoA activity is inhibited by several factors. The functions of Cdc42 are still unclear.

In the SVZ/IZ, nascent neurons acquire a multipolar morphology and then take on a bipolar morphology, which is believed to be critical for the progressive migration of nascent neurons to the CP [6,52,53]. Neurons are derived from RGPs or intermediate progenitors, which also originate from RGPs via INM, and migrate radially to the IZ [6,52,54]. Meanwhile, there are two populations of nascent neurons in the VZ/SVZ with distinct migratory behaviors (Figure 2A). One population is derived directly from RGPs and remains in the lower part of the SVZ, which is usually referred to as the multipolar cell accumulation zone. This population is termed the “slowly exiting population” (SEP) [52]. Meanwhile, the other population, termed the “rapidly exiting population” (REP), rapidly migrates into the SVZ/IZ and subsequently differentiates into intermediate progenitors, which can further divide and differentiate into multipolar neurons [52]. The SEP neurons reach the CP earlier than the REP neurons [52]. Nevertheless, both populations undergo a transient multi-bipolar period with no defined cell polarity for one day or more and then take on a bipolar morphology before they reach the CP [53,55]. During the multipolar–bipolar transition, the dynamic processes of the nascent neurons actively extend and retract. They may explore the microenvironment through the frequent generation of neurites in response to guidance factors and thus establish cell polarity in the proper direction [56]. Then, one of the multiple neurites elongates to form the leading process, while a thin axon appears in the opposite direction [52,55]. Afterward, the multipolar neurons acquire a bipolar morphology. Dynamic contacts between neurons and RGPs are established upon the multipolar–bipolar transition. The neurons subsequently navigate to the upper part of the CP by using these contacts as a scaffold. Defects in the multipolar–bipolar transition lead to the accumulation of migrating neurons in the lower IZ, ectopic lamination, and disturbed circuit formation, which may cause intellectual disability or epileptic seizures [57,58,59,60].

The multipolar–bipolar transition is a complex process regulated by the cooperative actions of extracellular and intracellular signals. One of the fundamental requirements for this transition is Rho GTPases, which link upstream signalings with cytoskeletal dynamics (Figure 2B). In addition, by regulating the cytoskeleton, Rho GTPases promote growth cone formation and retraction in neurites, supporting microenvironment exploration and thus the multipolar–bipolar transition. Besides growth cone formation and retraction, dynamic contacts with RGPs and cell polarity stabilization are also very important for multipolar stage exit. Rho GTPases might also participate in establishing these contacts. 

### 3.1. Role of Rac1 in the Multipolar–Bipolar Transition

Optimized Rac1 activity, i.e., a proper ratio of GTP-bound/GDP-bound Rac1, is required for the multipolar–bipolar transition of migrating neurons. Either constitutively active (CA) Rac1 or dominant-negative (DN) Rac1 results in migration defects that manifest as ectopic accumulation of multipolar neurons in the IZ [51,61]. These findings suggest that balanced Rac1 activity is required for the multipolar–bipolar transition, similar to the requirement of balanced Rac1 activity during the maintenance of neuromuscular acetylcholine receptor clusters [62].

There are several RacGEFs potentially involved in Rac1 activity regulation during the multipolar–bipolar transition. For example, P-Rex1, a RacGEF activated by autism susceptibility candidate 2 (AUTS2), regulates neuronal migration by augmenting Rac1 activity [57,58]. In addition, DOCK4, an atypical RacGEF from the DOCK family, can promote neurite differentiation through Rac1 activation [63], and the roles of DOCK–Rac1 signaling in developing neurons have been discussed extensively [2,50,64]. However, further investigation is needed to decipher the exact roles of these RacGEFs to modulate Rac1 function in the multipolar-bipolar transition. 

Besides RacGEFs, Rac1 activity can be regulated by other signalings. For example, robo4, a member of the robo family, which act as repulsive factors in tangential migration, is reported to facilitate radial migration through antagonizing slit/srGAP2 to activate Rac1 [65,66,67]. Rac1 can also be recruited to the plasma membrane by protocadherin to promote actin polymerization in neurites [68]. Another key upstream factor is transient axonal glycoprotein-1 (TAG1), which help sustain Rac1 activity in neurites for elongation [69]. Moreover, Rac1 can exert its roles by diverse downstream effectors. Rac1 forms a complex with Wiskott–Aldrich syndrome family verprolin-homologous protein complex (WAVE) to control actin polymerization [68,70]. In addition, Rac1 partially rescues the multipolar–bipolar transition in Ras-proximate-1 (Rap1)-inhibited neurons, implying that Rac1 may activate Rap1, which conveys the Reelin signal to N-cadherins to mediate neuronal polarization [7]. JNK acts downstream of Rac1 and plays a critical role in the multipolar stage exit by regulating microtubule dynamics via MAP1B and DCX [51,71]. These findings collectively suggest that Rac1 acts as a key signaling transducer that links extracellular or intrinsic factors with cytoskeletal dynamics and thus promotes the multipolar–bipolar transition. 

Interestingly, deletion of Rac1 in the VZ only delays the onset of neuronal migration rather than blocking the process like CA Rac1 or DN Rac1 [51,61]. These findings suggest a possible compensatory mechanism by which other related Rho GTPases and/or signaling pathways overcome the loss of Rac1. This hypothesis is supported by the finding that migrating neurons maintain their cell polarity through P-Rex1/Rac3 [72]. It is also noteworthy that some RacGAPs may regulate the multipolar–bipolar transition independent of Rac1 activity. For example, α2-chimaerin, a RacGAP, promotes the multipolar–bipolar transition via its SH2 domain to modulate a microtubule-associated protein, CRMP-2, rather than through direct interaction with Rac1 via its GAP domain [59]. 

### 3.2. Role of Cdc42 in the Multipolar–Bipolar Transition

Unlike Rac1, which is ubiquitously expressed in the developing cortex, Cdc42 expression is concentrated in the VZ and CP [73], implying their differential roles and underlying mechanisms in neuronal migration. In the developing cortex, the introduction of DN Cdc42 or CA Cdc42 results in a small number of neurons arrested in the lower IZ, while DN Rac1 or CA Rac1 leads to a large number of neurons accumulating in the lower IZ [61]. Although Cdc42 is required to establish neuronal polarity, evidence to support its function during the multipolar–bipolar transition is still lacking [74,75,76]. Intriguingly, a recent study found that Cdc42-deficient granule cell precursors possess a multipolar morphology in the external germinal layer rather than in the cortex, indicating that Cdc42 may play a critical role in neuronal polarity in a region-specific manner [77].

### 3.3. Role of RhoA Inhibition in the Multipolar–Bipolar Transition

RhoA is required for the maintenance of the cell polarity of RGPs, and RhoA expression decreases dramatically when nascent neurons reach the IZ [73]. Consistent with this observation, Arhgef1, a RhoGEF that inhibits neurite outgrowth via the RhoA/ROCK pathway, is downregulated in newly born neurons compared to RGPs [78,79]. Moreover, transplanted RhoA-knockout neurons migrate normally in vivo in a wild-type environment with normal RGP scaffolding, suggesting that intrinsic RhoA activity is not required for the migration process [33]. Interestingly, when the CA form of RhoA is introduced into migrating neurons, the multipolar–bipolar transition is impaired [80]. Indeed, RhoA activity is inhibited by cyclin-dependent kinase 5 (Cdk5), which acts as a central player in neuronal migration [80,81,82]. For instance, during the multipolar–bipolar transition, Mammalian Ste20-like kinase 3 (Mst3), whose activity is dependent on Cdk5, downregulates RhoA activity. Meanwhile, Mst3 deficiency results in upregulated RhoA activity and thus the arrest of neurons in the lower IZ with a multipolar morphology [80]. Furthermore, p27kip1, another substrate of Cdk5, is also required for multipolar stage exit by regulating cytoskeletal dynamics through inhibiting RhoA activation and promoting cofilin activity [83,84,85]. Therefore, the inhibition of RhoA activity is thought to be crucial for the multipolar–bipolar transition. 

It is notable that RhoA activity is regulated in the opposite direction of Rac1 or Cdc42 activity. When Rac1 or Cdc42 is recruited to the extension growth cone, RhoA activity is inhibited [86,87]. In neurons, RhoA activation coupled with Rac1 or Cdc42 inactivation causes neurite retraction due to increased actomyosin contractility via Rho kinase (ROCK)/myosin light chain (MLC) [86,87]. Meanwhile, LIM kinases are serine/threonine kinases that can be activated by ROCK and thus inhibit the actin depolymerization activity of cofilin in neurons [86,87]. Balanced activity between RhoA and Rac1/Cdc42 ensures efficient multipolar–bipolar transition and migration.

### 3.4. Role of Rnd2-Mediated RhoA Inhibition in the Multipolar–Bipolar Transition

In migrating neurons, Rnd2 is expressed transiently during a brief “stopover” in the SVZ and IZ [73]. Rnd2-deficient neurons accumulate in the IZ and exhibit a multipolar morphology with long processes, indicating that Rnd2 activity is required for multipolar stage exit [12,14,16,88,89]. Rnd2 functions as a CA form and is regulated primarily by its expression level [89,90]. Rnd2 expression can be regulated by a series of transcription factors such as Neurog2, COUP-TFI, and RP58. Neurog2, a proneural transcription factor, upregulates Rnd2 expression, whereas COUP-TFI and RP58 downregulate Rnd2 expression [12,14,15]. Rnd2 downregulates RhoA function by either antagonizing RhoA activity through the stimulation of p190RhoGAP or promoting RhoA degradation through a Rnd2-binding protein, BTB-domain containing adaptor for Cul3-mediated RhoA degradation 2 (Bacurd2) [13,88]. 

However, there might be other unidentified processes by which Rnd2 inhibits RhoA. Accordingly, migration defects due to Rnd2 silencing can be rescued by overexpression of a DN form of Rnd2 rather than a CA form of cofilin [16], which strongly supports the presence of actin-independent Rnd2 function during the migration. 

## 4. Locomotion

**Figure 3:**
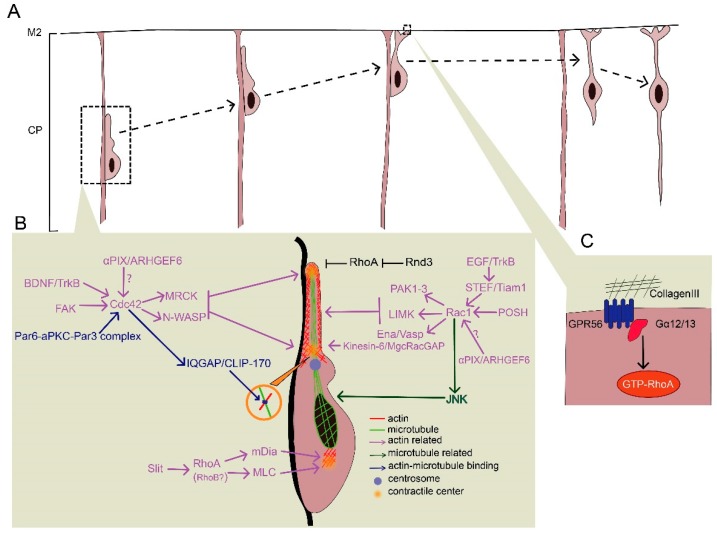
Rho GTPases regulate locomotion and terminal translocation. (**A**) Neurons migrate radially along RGPs until they reach the marginal zone and receive a stop signal to uncouple from RGPs at their final destinations. (**B**) In the locomotion process, leading process and dilation formation rely on the proper distribution of activated Rac1. Rac1 regulates both actin (red) and microtubule (green) dynamics through distinct pathways. In addition, RhoA activity should be inhibited during locomotion; Rnd3 is a key inhibitory factor of RhoA. Pulling force is generated by three contractile centers under the regulation of Cdc42 and RhoA. Cdc42 mainly functions in the two contractile centers localized at the distal and proximal regions of the leading process, while RhoA contributes to retraction of the trailing process. Most factors (red) regulate actin dynamics through non-muscle myosin-II, while some factors (blue) help tie microtubule ends with the actin cytoskeleton. (**C**) RhoA transduces stop signals to migrating neurons in terminal translocation. Upon interacting with collagen III, RGP56 couples to Gα12/13 and thus activates the RhoA pathway.

After exiting the multipolar stage, neurons moving towards the CP exhibit a characteristic bipolar morphology with a long leading process and a short trailing process towards the pial surface and VZ, respectively. These neurons undergo a unique process called locomotion (Figure 3A), which is divided into four steps: (1) The formation and extension of a leading process, (2) the formation of proximal cytoplasmic dilation in the leading process, (3) somal translocation, and (4) the retraction of the trailing process [91]. The leading process is thought to guide the migration by responding to various chemoattractants or chemorepellents. The cell soma remains largely static when the leading process explores the environment. However, after the selected leading process is stabilized, the translocation of the centrosome occurs, followed by nuclear translocation and trailing process retraction. During the locomotion process, migrating neurons use RGPs as scaffolds, and more importantly, a dilation forms at the proximal region in the leading process, followed by centrosome nuclear translocation [92,93,94]. This cytoplasmic dilation contains the centrosome, Golgi apparatus, and microtubules [91,94]. Cytoskeletal rearrangement in distinct domains of migrating neurons, such as the leading process, cytoplasmic dilation, and perinuclear space, are tightly regulated by a coordinated network of signaling pathways. Therefore, they ultimately lead to oriented locomotion. Both the actin and microtubule cytoskeleton are involved in this multistep process. Locomotion failures may lead to subcortical laminar heterotopia or lissencephaly, with epilepsy and cognitive impairment [95]. These severe symptoms result from the dysregulation of both the actin and microtubule cytoskeletons accompanied by disturbed centrosome-nuclear coupling and impaired generation of pulling force [95,96,97].

### 4.1. Formation of the Leading Process and Dilation

Secreted guidance cues, extracellular matrix, and cell-surface guidance cues—chemoattractive or chemorepulsive—play essential roles in the formation of the leading process and dilation, many of which convey the signal for cytoskeletal reorganization via Rho GTPases (Figure 3B).

#### 4.1.1. Role of Rac1 in the Formation of the Leading Process and Dilation

Although Rac1 is expressed throughout the VZ and IZ, the finding that some Rac-specific GEFs exhibit specific localization might provide new insights into the spatiotemporal regulation of Rac1 during the formation of the leading process and cytoplasmic dilation. For instance, the restricted localization of STEF/Tiam1 (a Rac-specific GEF) in the migrating neurons in the IZ and CP indicates that Rac1 is specifically activated during the locomotion process, which differs from its contribution to RGP proliferation in the VZ [43,44,98]. STEF/Tiam1 plays an essential role in locomotion by regulating leading process formation. The DN form of STEF/Tiam1 results in stalling neurons being arrested in the IZ without differentiating [51]. One possible explanation is that when Rac1 activity is inhibited by STEF/Tiam1 disruption, the outgrowth and stability of neurites are disturbed, and thus the leading process fails to form [99,100,101]. Furthermore, STEF/Tiam1 can be recruited by the neurotrophic receptor, TrkB, to the membrane to promote lamellipodia formation [101]. TrkB can be activated by epidermal growth factor (EGF) in radial migration rather than by brain-derived neurotrophic factor (BDNF) or neurotrophin-4 in tangential migration [102,103,104]. These results indicate that an EGF–TrkB–STEF/Tiam1–Rac1 axis conveys the message from extracellular signals to cytoskeletal dynamics in leading process formation. Another Rac/Cdc42 GEF involved in locomotion is αPIX/ARHGEF6. Unlike STEF/Tiam1 in leading process, αPIX/ARHGEF6 activates Rac1 or Cdc42 in centrosome translocation during cytoplasmic dilation in migrating hippocampal neurons [105]. MgcRacGAP, a RacGAP concentrated in the proximal region of the leading process, regulates actin dynamics and thus limits redundant protrusion to maintain a single leading process, although it is unclear if Rac1 acts downstream to elicit the signals to the cytoskeleton [64,106]. The Rac activator, DOCK7, plays an essential role in leading process extension and somal translocation through the regulation of both actin and microtubule dynamics during tangential migration [107]. DOCK7 is also required for the establishment of neuronal polarity by controlling axon formation [108]. However, the involvement of DOCK7 in the formation of the leading processing during radial migration awaits further investigation.

Plenty of SH3s (POSH) is a scaffold protein that is predominantly expressed in the leading process of the locomoting neurons [109]. Knockdown of POSH disturbs the formation of the proximal cytoplasmic dilation in the leading process, which subsequently disrupts centrosome translocation, nuclear translocation, and neuronal migration. This is accompanied by aberrant localization of activated Rac1 and impaired F-actin assembly [109]. Thus, it is likely that activated Rac1 is recruited to the cell membrane by POSH to stabilize the leading process and dilation. One effector downstream of Rac1-POSH is p21-activated kinases (PAK1-3), which can be activated by Rac1 and thus promote actin polymerization via the LIMK-cofilin pathway [110]. In addition, Ena/Vasp, another downstream effector of Rac1, regulates neuron positioning but not morphology during neuronal migration via actin assembly [111,112]. Therefore, Rac1 is able to regulate actin dynamics to promote the maintenance of the leading process during neuronal locomotion. 

#### 4.1.2. Effects of RhoA Inhibition on Leading Process and Dilation Formation

In contrast to the action of Rac1, RhoA inactivation appears to be critical for migrating neurons. Activated RhoA causes actomyosin contractility via ROCK/MLC to induce growth cone collapse, thus inhibiting the formation of the leading process [113]. During locomotion, RhoA activity can be inhibited by various cell-autonomous and extrinsic factors. The loss of some receptors results in impaired RhoA inhibition. For example, cannabinoid CB1 receptor knockdown increases RhoA activity and impairs neuronal migration [114]. In migrating neurons, RhoA expression is also transcriptionally repressed by histone modification proteins such as chromodomain Y-like (CDYL), which is reported to be critical for neuronal migration [60]. In addition, RhoA activity can be downregulated by RhoGAPs such as p190RhoGAP and srGAP2, which are regulated by certain proneural transcription factors including Neurog1 and Neurog2 [16]. 

#### 4.1.3. Rnd3-Mediated RhoA Inhibition in Leading Process and Dilation Formation

Rnd proteins act as inhibitory factors of RhoA during migration. As described earlier, Rnd2 inhibits RhoA activity during the multipolar–bipolar transition, whereas Rnd3, another member of the Rnd protein family, downregulates RhoA activity primarily during locomotion [88,89,90,115]. Rnd3 localized at the plasma membrane links the signals from RGP contacts and the cytoskeleton [88]. Ascl1, a transcription factor with a basic helix–loop–helix structure, is thought to upregulate Rnd3 expression and thus inhibit RhoA activity to facilitate the locomotion process [16,51]. In addition, Semaphorin/Plexin B2 and Rnd3 antagonize each other’s activity and thus regulate RhoA activity in the leading process in a region-specific manner [88]. Interestingly, loss-of-function studies show that Rnd3 knockdown results in inflated leading processes with multiple thin processes [88]. Rnd3-deficient neurons exhibit an apparently increased number of branches in the leading process probably due to the failure of establishing proper contacts with RGPs [16,88,115]. Knockdown of RhoA rescues the migration defects caused by Rnd3 silencing, suggesting that Rnd3 promotes locomotion through RhoA inhibition [88]. In Rnd3-silenced neurons, there is an increased distance from the nucleus to the centrosome, indicating disturbed nuclear–centrosome coupling [88]. This abnormality may be due to impaired actin depolymerization, as overexpression of a non-phosphorylatable form of cofilin rescues this defect in RGPs [51]. 

### 4.2. Maintenance of Proper Contractile Activity

In migrating neurons, proper contractile activity is indispensable for nucleokinesis (Figure 3B). At the subcellular level, the contractile activity is primarily provided by non-muscle myosin-II [97,116]. The contraction force might be generated simultaneously at three distinct regions in migrating neurons rather than at just one contractile center as was previously thought: Two contractile centers are localized at the distal and proximal region of the leading process, respectively, and the third is in the trailing process [117]. Furthermore, a pulling force rather than a pushing force is believed to be responsible for nucleokinesis and the retraction of the trailing process [117]. This force, generated at the three contraction centers is dynamically regulated by various cytoskeletal components and adhesions.

#### 4.2.1. Cdc42-Mediated Contraction in the Leading Process

BDNF, slit homolog 2 protein (Slit2), and the focal adhesions act as extracellular factors to mediate force generation at contraction centers [102,118,119,120,121]. Specifically, Cdc42 acts downstream of BDNF and the focal adhesion, and RhoA acts downstream of slit2. Cdc42 primarily influences contraction centers at the leading process, whereas RhoA plays a role in the retraction of the trailing process, which will be discussed in Section 4.2.2. [117]. Focal adhesions may mediate the modulation of Cdc42 through cytosolic factors such as focal adhesion kinase (FAK) and thus regulate contraction center activity via cytoskeletal regulation [86,120]. BDNF might mediate the modulation of Cdc42 via binding membrane receptors such as TrkB [103]. In addition, the Par6/aPKC/Par3 polarity protein complex can recruit Cdc42 and thus regulate cytoskeletal dynamics to maintain neuronal polarity for locomotion [122,123,124]. 

The activation of Cdc42 further promotes myosin-II activity through modulating myotonic dystrophy-related Cdc42-binding kinases (MRCK) and also promotes F-actin polymerization via the N-WASP/Arp2/Arp3 pathway [125,126,127]. In addition, Cdc42 regulates microtubule dynamics by forming Cdc42/IQGAP1/CLIP-170 tripartite complex, which ties microtubule ends with the actin cytoskeleton [96,128]. Thus, both actin and microtubule dynamics are under the control of Cdc42 regulation to promote myosin-II–driven architecture construction, which generates pulling force.

#### 4.2.2. RhoA-Mediated Formation and Retraction in the Trailing Process

The formation and retraction of the trailing process are characteristic features of radial migration. The concentration of myosin-II motor is high in the leading process, where it initiates the retraction of the trailing process [97,129,130]. Unlike the action of Cdc42, RhoA contributes to the retraction of the trailing process by shifting the dominant contraction centers of the leading and trailing processes [117]. In migrating neurons, frontal administration of Slit2, which is believed to act as a repulsive factor for neuronal migration, reverses the distribution of RhoA towards the trailing process, resulting in the shift of the leading–trailing process [67,117,131,132,133]. 

The rearrangement of RhoA can facilitate myosin-II activation through ROCK, which augments MLC activity by activating MLC and inactivating MLC phosphatase (MLCP) by phosphorylation [92]. In addition, RhoA can act on mDia1, an actin nucleator, to drive actin polymerization and promote contraction [134,135]. It should be noted that most supporting evidence for the multiple contraction points model comes from in vitro studies. Moreover, related signaling molecules, such as BDNF, slit, and mDia1, are reported to play essential roles in tangential migration rather than radial glial migration. 

As discussed above, cell-autonomous RhoA activity is not required for neuronal migration, as transplanted RhoA-deficient neurons migrate normally by using wild-type RGPs as a scaffold [33]. Even though the downregulation of RhoA activity is required for neuronal migration, restricted low levels of RhoA might exist in microregions to regulate actomyosin contractility. This notion is supported by the fact that RhoA appears to be present in the CP at later stages when most migrating neurons adopt a locomotion mode [136]. Alternatively, compensatory expression of RhoB might take the place of RhoA. This is supported by the fact that RhoA is enriched in the VZ where RGPs remain, whereas RhoB is mainly expressed in the CP where neurons undergo migration [73]. In addition, in the absence of RhoA, RhoB expression appears to increase in order to compensate [137]. Thus, RhoB might be a substitute for RhoA activity to generate pulling force during neuronal locomotion. 

## 5. Terminal Translocation

At the final stage of neuronal migration, once the leading process reaches the marginal zone, neurons need to stop upward locomotion and shift to migrating a short distance independent of the radial glial fibers. This migration mode is called terminal translocation (Figure 3A). It is fundamental for migrating neurons to receive stop signals. If neurons fail to transduce stopping signals to cytoskeletal dynamics, excess neuronal migration occurs [138]. Neuronal over-migration breaches the pial membrane and also causes defects in neuron positioning. One such defect is cobblestone lissencephaly, which is characterized by the disruption of pial basement membrane integrity [36,38,138].

It is clear that the Rab7-dependent lysosomal degradation of the N-cadherin and reelin–integrin α5β1 pathway contributes to the normal terminal translocation process, but less is known about the regulation of Rho GTPases in terminal translocation [8,139]. However, several studies have revealed that RhoA also plays a role in terminal translocation (Figure 3C) [37,111]. Given that RhoA is expressed in the CP at later stages when terminal translocation occurs, it might convey extracellular stop signals to slow down neuronal migration [136]. For example, G protein-coupled receptor 56 (GPR56), a member of the adhesion GPCR family, is activated through interaction with its ligand, collagen III, and couples to the Gα12/13 protein to activate the RhoA pathway [111]. Upon GPR56 knockout, the pial membrane breaks down concurrently with neuronal over-migration, which is due to the inactivation of RhoA [37,111]. One possibility is that RhoA activation helps result in leading process retraction through ROCK activation and thus uncouples the connection between neurons and RGPs during terminal translocation [113,140,141]. However, it remains unclear how RhoA regulates terminal translocation and whether some other Rho GTPases are involved.

## 6. Perspectives

To build the well-organized six-layered architecture of a functional cortex, neurons go through a migration process that is tightly controlled by extracellular or intrinsic signals. Rho GTPases serve as the key bridge linking extracellular or intrinsic signals to regulate cytoskeletal dynamics in a spatiotemporal-specific manner during neuronal migration (Table 1). However, there are several important questions to be explored in order to clarify the molecular basis that underlies the action of Rho GTPases in this process. Although the maintenance of adherens junctions between RGPs is required to support neuronal migration, how adhesion junctions convey signals to the cytoskeleton via Rho GTPases remains largely unknown. It would be interesting to investigate the signaling cascades by which the key protein complexes in adherens junctions directly or indirectly control the activity of Rho GTPases, as well as their corresponding GEFs or GAPs. In parallel, an outstanding challenge is understanding how distinct extracellular factors and their expression gradients are coordinated to transduce signals to specific Rho GTPases at specific stages during the migration process as well as how the optimal activities of multiple Rho GTPases are coordinated simultaneously and within the same neuron. Moreover, the potential compensatory roles of different Rho GTPases are still not well characterized. Future investigations of the migration process at the single-neuron level will definitely shed light on the mechanisms that underlie cortical development and related brain disorders.

## Figures and Tables

**Table 1:** Rho GTPases regulate different processes during neuronal migration.

Stage	Rho GTPase	Role
Neurogenesis (INM, in RGPs)	Cdc42	Mediates adherens junction formation, establishes apical polarity, promotes RGP self-renewal
	RhoA	Maintain adherens junctions, balances proliferation and differentiation of RGPs
	Rac1	Promotes basal–apical nuclear movement, regulates cycle exit
	Rnd3	Promotes basal–apical nuclear movement, mediates apical attachment of RGPs and cleavage plane orientation
Multipolar–bipolar transition	Rac1	Promotes neurite elongation
	Cdc42	Establishes neuronal polarity
	RhoA	Entirely inhibited
	Rnd2	Inhibits activity of RhoA
Locomotion	Rac1	Promotes the formation of the leading process and dilation
	RhoA	Entirely inhibited in leading process; Mediates retraction in the trailing process
	Cdc42	Mediates contraction of the leading process
	Rnd3	Inhibits activity of RhoA
Terminal translocation	RhoA	Stops over-migration
